# Hemolytic Streptococcus May Exacerbate Kidney Damage in IgA Nephropathy through CCL20 Response to the Effect of Th17 Cells

**DOI:** 10.1371/journal.pone.0108723

**Published:** 2014-09-29

**Authors:** Ting Meng, Xiaozhao Li, Xiang Ao, Yong Zhong, Rong Tang, Weisheng Peng, Jinghua Yang, Mingxiang Zou, Qiaoling Zhou

**Affiliations:** 1 Department of Nephrology, Xiangya Hospital, Central South University, Changsha, Hunan, China; 2 Department of Clinical Laboratory, Xiangya Hospital, Central South University, Changsha, Hunan, China; Center for Molecular Biotechnology, Italy

## Abstract

**Background:**

The exacerbation of IgA nephropathy (IgAN) is related to respiratory tract infection with hemolytic streptococcus (HS), but the mechanism is unknown. In this study we investigated the role of chemokine ligand 20 (CCL20) in response to the effect of T helper 17 (Th17) cells in the pathogenesis of IgAN associated with HS.

**Methods:**

Thirty mice were randomly divided into five groups: control mice (control), IgAN mice (IgAN), HS-infected IgAN mice (HS-IgAN), CCL20-treated IgAN mice (CCL20-IgAN), and CCL20-treated HS infected IgAN mice (CCL20-HS-IgAN). IgAN mice were induced with lipopolysaccharide, carbon tetrachloride and bovine serum albumin. Then the mice were sensitized with CCL20 antibody and infected with alpha-hemolytic streptococcus (α-HS) isolated from tonsils in sequence. Urine Albumin-Creatinine ratio and sediments were measured. The pathological changes in kidney and lung tissues were observed under microscopy. Th17 cells and regulatory T cells (Tregs) in kidneys were tested by flow cytometry. CCL20, IL-17A, IL-6 and IL-21 in the kidneys were detected by ELISA.

**Results:**

The IgAN mice had albuminuria and microscopic hematuria, renal mesangial proliferation, IgA deposition, high electron dense deposition in glomerular mesangial region, decreased frequency of Tregs, increased frequency of Th17 and Th17-Treg ratio. Furthermore, Th17-related cytokines CCL20, IL-17A, IL-6 and IL-21 were all increased in the kidneys of IgAN mice. Compared with IgAN mice, the manifestations in HS-IgAN mice were more severe, but alleviated in CCL20-treated groups.

**Conclusion:**

α-HS may exacerbate kidney damage in IgAN through CCL20 response to the effect of Th17 cells.

## Introduction

IgA nephropathy (IgAN) is the most common primary glomerulonephritis throughout the world, and is more common in younger adults. About 20%–30% of IgAN cases progress to chronic renal failure in a period of 20 years [1], and it is an important cause of end-stage renal disease (ESRD) [2]. IgAN is always exacerbated after an upper respiratory tract infection with hemolytic streptococcus (HS), with α-HS being a common type [3].

CD4^+^ T-helper cells are important mediators involved in the development of glomerulonephritis [4]. Th17 cells are a subgroup of T-helper cells producing interleukin 17 (IL-17), which plays role in inflammation and tissue injury. As a new lineage of effector T-helper cells, there is increasing evidence implicating Th17 cells in nephritis, asthma and other autoimmune diseases [5], [6], [7], [8]. Wang B et al. demonstrated that HS infection can induce the differentiation of Th17 cells [9], but whether it is involved in the pathogenesis of IgAN or not remains unknown.

Accumulative evidence indicates that infiltrating Th17 cells secrete IL-17, which stimulates resident renal cells through some receptors to produce CCL20. CCL20 is a small cytokine that can attract lymphocytes, neutrophils, monocytes and dendritic cells toward epithelial cells. CCL20 and the unique receptor CCR6 might be involved in the recruitment of T cells to organize nodular infiltrates in chronic renal inflammation. CCL20 interacts with the corresponding receptor CCR6, which leads to the recruitment of pro-inflammatory leukocyte subsets (neutrophils, lymphocytes, etc) and ultimately leads to the progression of immune-mediated kidney damages [10]. In view of the relationship between CCL20 and Th17, this study was designed to clarify whether hemolytic streptococcus-exacerbated kidney damage in IgAN is involved in the CCL20 response to the effect of Th17 cells.

## Results

### HS increases and CCL20 antibody decreases albumin-to-creatinine ratio (ACR) in IgAN mice

**Figure 1:**
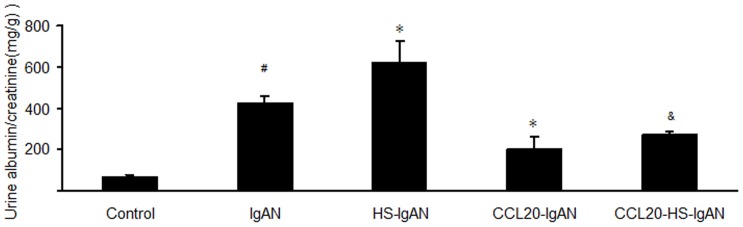
HS increases and CCL20 antibody decreases ACR in IgAN mice. ^#^ *vs* control group, *P*<0.05; ^*^ *vs* IgAN group, *P*<0.05; ^&^ *vs* HS-IgAN group, *P*<0.05.

To determine the negative effect of HS and beneficial effect of CCL20 antibody on kidney injury in IgAN mice, urine samples of all the mice were collected for the assessment of ACR at the end of the 11^th^ week after administration of HS and CCL20 antibody. We found that the ACR was significantly elevated in IgAN mice compared with controls (427.90±28.43 mg/g *vs* 66.57±7.99 mg/g, *P*<0.05). This indicates that we have successfully established an IgAN mouse model. Administration of HS further significantly increased the ACR in HS-IgAN vs. IgAN mice (625.88±97.49 mg/g *vs* 427.90±28.43 mg/g, *P*<0.05). In contrast, treatment of the IgAN mice with CCL20 antibody rescued the phenotype, as evidenced by a significant reduction of the ACR from 427.90±28.43 mg/g in IgAN group to 202.93±58.86 mg/g in the CCL20-IgAN group. Co-administration of both HS and CCL20 antibody yielded an intermediate level of the ACR between HS-IgAN and CCL20-IgAN. This intermediate level was still significantly lower than the ACRs of both IgAN and HS-IgAN groups (Figure 1). Taken together, our data suggest that HS significantly increases and CCL20 antibody significantly decreases ACR in IgAN mice. CCL20 antibody can effectively antagonize the detrimental effect of HS on the renal function evaluated by ACR.

### HS worsen and CCL20 antibody decreases renal damage in IgAN mice

**Figure 2:**
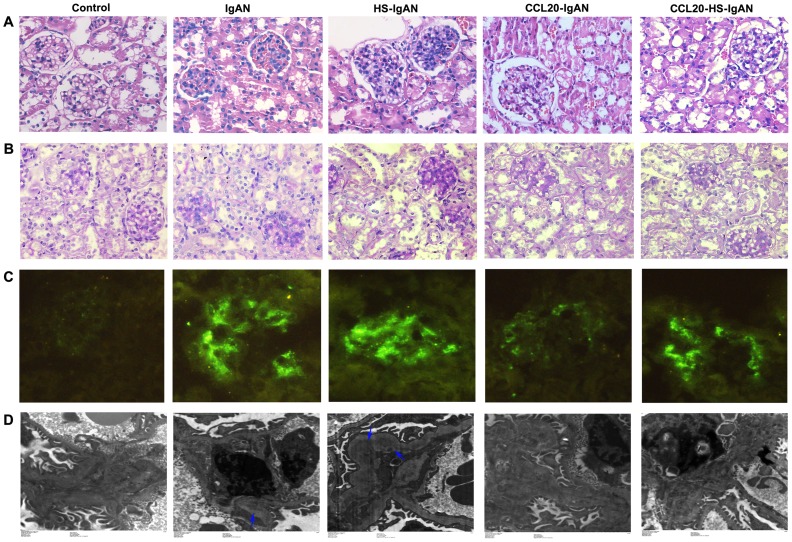
HS worsens and CCL20 antibody improves renal damage in IgAN mice. Representative images of HE-stained (A, 400×), PAS-stained (B, 400×), Immunofluorescence (C, 200×) and transmission electron micrographs (D) kidney sections from mice as indicated. For immunofluorescence staining, IgA antibody was used. The arrows in D point to high electron dense deposition in glomerular mesangial region.

To evaluate the kidney damages, we first examine Hematoxylin and Eosin (HE)-stained kidney sections to determine the histological changes. We found that, IgAN mice had pronounced proliferation of the mesangium compared with the control mice. The proliferation was exacerbated in the HS-IgAN mice, but ameliorated in CCL20-treated mice (Figure 2A). Similar results were obtained when periodic acid–Schiff (PAS)-stained sections of kidneys from these mice were examined (Figure 2B). Immunofluorescence staining with an antibody specific for IgA revealed IgA deposition in the IgAN group. The IgA-specific fluorescence signal became stronger in the HS-IgAN group, and weaker in CCL20-treated groups (Figure 2C). The changes in histology and IgA deposition were associated with corresponding changes in the electron dense deposition, which were unearthed by electron microscopy. More specifically, we observed a high electron dense deposition in the glomerular mesangial region of IgAN mice. The electron dense deposition was enhanced in the HS-IgAN group and reversed in the CCL20-treated groups (Figure 2D). There was no mesangial proliferation, IgA deposition and electron dense deposition in the control group. In each case, the CCL20-HS-IgAN group displayed an intermediate phenotype between HS-IgAN and CCL20-IgAN, consisting with what we observed for the ACR. Our morphological results suggest that HS worsens and CCL20 antibody improves renal damage in IgAN mice.

**Figure 3:**
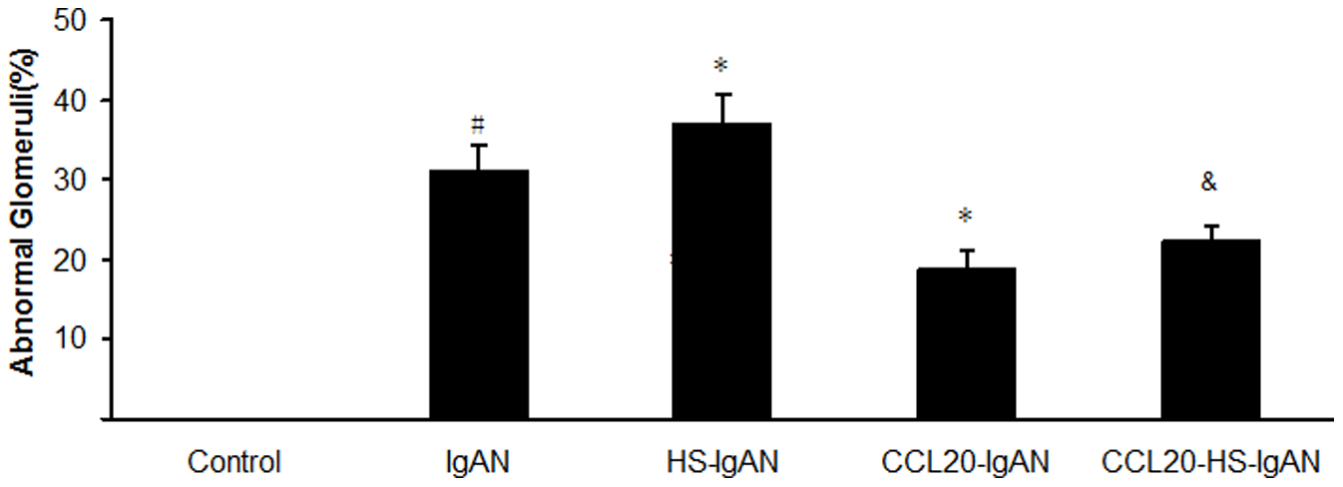
HS increases and CCL20 antibody decreases abnormal glomeruli in IgAN mice. PAS-stained kidney sections were evaluated for the presence of abnormal glomeruli. ^#^ *vs* control group, *P*<0.05; * *vs* IgAN group, *P*<0.05; ^&^ *vs* HS-IgAN group, *P*<0.05.

To more accurately quantify the lesions in the kidneys, the presence of abnormal glomeruli (AG) was evaluated by PAS-staining (Figure 3). The frequency of AG at week 11 was significantly increased in IgAN vs. controls (31.2±3.0% *vs 0.0*±0.0%; *P*<0.05) and in HS-IgAN vs. IgAN mice (37.2±3.3% *vs* 31.2±3.0%; *P*<0.05). However, CCL20-IgAN and CCL20-HS-IgAN mice showed significantly decreased frequency of AG compared with IgAN mice and HS-IgAN mice, respectively (18.8±2.3% *vs* 31.2±3.0%, *P*<0.05; 22.4±1.7% *vs* 37.2±3.3%, *P*<0.05). The quantitative results of kidney injury are consistent with morphological results.

### HS worsen and CCL20 antibody decreases lung damage in IgAN mice

**Figure 4:**
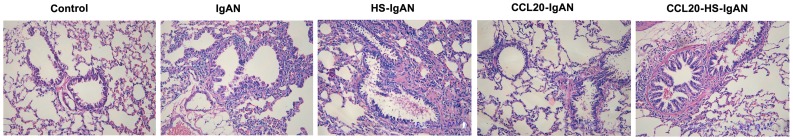
HS worsen and CCL20 antibody improves lung damage in IgAN mice. Representative images of HE stained lungs from mice as indicated (200×).

To investigate the effect of HS and CCL20 antibody on airway inflammation of IgAN mice, we examined HE-stained lung sections of the mice from each group. Compared with the control mice, IgAN and HS-IgAN mice had a remarkable infiltration of inflammatory cells surrounding the airways. These pathological changes were alleviated in the CCL20-treated mice (Figure 4). Hence, like in kidney, HS deteriorates and CCL20 antibody ameliorates the injury in lung characterized by airway inflammation in IgAN mice.

### HS increases and CCL20 antibody decreases Th17/Treg in IgAN mice

**Figure 5:**
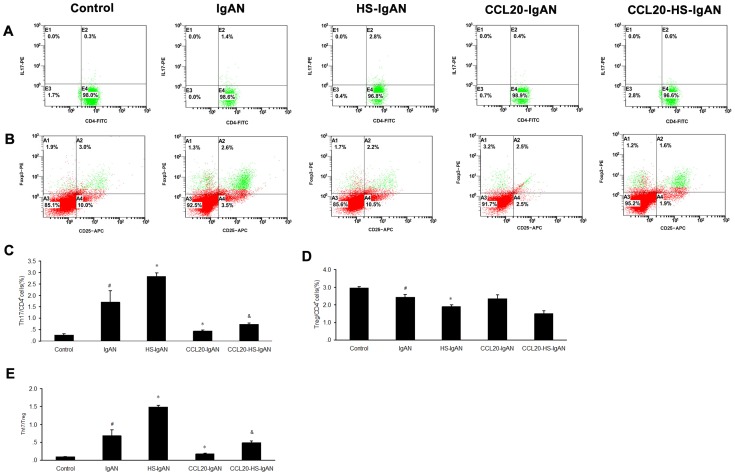
HS increases and CCL20 antibody decreases Th17/Treg in IgAN mice. Isolated leukocytes were analyzed by flow cytometry. For production of IL-17 and Foxp3, numbers are counts in percentage of CD4^+^T cells. Collective analysis of the results from each group (C, D, E). ^#^ *vs* control group, *P*<0.05; ^*^ *vs* IgAN group, *P*<0.05; ^&^ *vs* HS-IgAN group, *P*<0.05.

To characterize T cell response in the kidneys, we separated lymphocytes from kidneys of mice at week 11 after treatment with HS and/or CCL20 antibody and analyzed them by flow cytometry. IgAN mice showed a substantially increased percentage in Th17 cells as compared with control mice (1.7±0.5% *vs* 0.27±0.06%, *P*<0.05). The frequencies of Th17 cells were further significantly increased to 2.8±0.15% in the HS-IgAN group, and significantly reduced to 0.43±0.06% in the CCL20-IgAN group. The number of Th17 cells in CCL20-HS-IgAN group (0.73±0.06%) lies between those of HS-IgAN and CCL20-IgAN groups (Figure 5A, 5C).

The frequencies of Treg cells were markedly decreased in the IgAN mice compared with control mice (2.6±1.6% *vs* 3.0±0.06%, *P*<0.05), and an even lower frequency was found in HS-IgAN mice compared with IgAN mice (1.9±0.1% *vs* 2.6±1.6%, *P*<0.05). However, there was no statistical difference between CCL20-IgAN mice and IgAN mice in the percentage of Treg cells (2.4±0.2% *vs* 2.6±1.6%), and a similar result was found between CCL20-HS-IgAN mice and HS-IgAN mice (1.5±0.2% *vs* 1.9±0.1%) (Figure 5B, D).

The Th17 to Treg ratios of IgAN mice were significantly elevated compared with controls (0.7±0.2 *vs* 0.1±0.0, *P*<0.05), and HS-IgAN mice had a higher ratio compared with IgAN mice (1.5±0.0 *vs* 0.7±0.2, *P*<0.05). The Th17 to Treg ratio of CCL20-IgAN mice was decreased compared with IgAN mice (0.2±0.0 *vs* 0.7±0.2, *P*<0.05) and a similar result was found between CCL20-HS-IgAN mice and HS-IgAN mice (0.5±0.1 *vs* 1.5±0.0, *P*<0.05) (Figure 5E).

### HS increases and CCL20 antibody decreases Th17-related cytokines in IgAN mice

**Figure 6:**
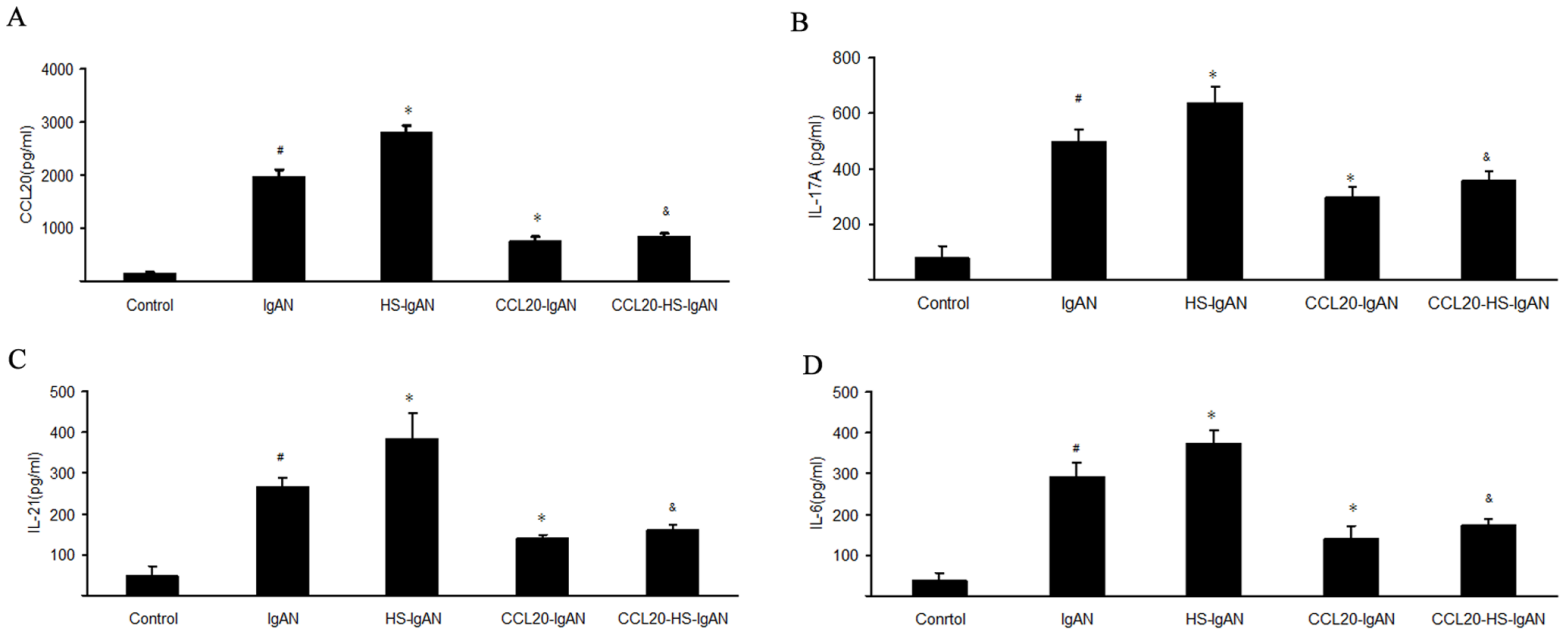
HS increases and CCL20 antibody decreases Th17-related cytokines in IgAN mice. (A): Renal CCL20 concentrations of each groups; (B): Renal IL-17A concentrations of each groups; (C): Renal IL-21 concentrations of each groups; (D): Renal IL-6 concentrations of each groups. ^#^ *vs* control group, *P*<0.05; ^*^ *vs* IgAN group, ^&^ *vs* HS-IgAN group *P*<0.05.

Previous studies have demonstrated that CCL20, IL-17A, IL-21 and IL-6 are all Th17-related cytokines. To verify if HS and CCL20 antibody affect Th17 cells, we performed Enzyme-Linked Immunosorbent Assay (ELISA) to determine CCL20, IL-17A, IL-21 and IL-6 in the kidneys in each group. The CCL20, IL-17A, IL-21 and IL-6 concentrations were all significantly higher in IgAN group than in the control group (Figure 6). The elevations of these cytokines were more dramatic in HS-IgAN group. This trend was reversed by the treatment with CCL20 antibody, as evidenced by a significant reduction of these cytokines in CCL20-IgAN vs. IgAN mice and in CCL20-HS-IgAN mice vs. HS-IgAN mice (Figure 6).

## Discussion

The exacerbation of IgAN is always associated with upper respiratory tract infection, and evidence has suggested that HS is the main bacterial strain isolated from tonsillar crypts [11]. Here we try to clarify the mechanism of α-HS infection exacerbating kidney damage in IgAN. Some researches have attempted to develop animal models for IgAN based on induced immune response [12], [13], [14]. Based on these studies, we produced the IgAN mice and infected them with α-HS isolated from human tonsils. Our model displayed the primary pathological features of human IgAN. These features include proliferation of mesangial cells expansion in mesangial matrix, and mesangial electron-dense deposits. The pathological changes in the kidneys of HS-IgAN mice were more severe, suggesting that α-HS infection lead to the exacerbation of IgAN. Furthermore, HS-IgAN mice had a remarkable infiltration of inflammatory cells surrounding the airway, which support the notion that α-HS induced immune responses cause the progression of immune-mediated kidney damage to respiratory tract infection.

The past decade of research has revealed an autoimmune nature of IgAN. The recruitment of T cells into the kidney is a main feature of glomerulonephritis. CD4^+^ T-helper cells have a key role in the regulation of immune response. Th17 and Tregs are new subsets of these CD4^+^ T cells [15]. Tregs suppress self-reactive T cells and maintain immunologic self-tolerance [16]. The feature of Th17 cells is the expression of IL-17. IL-17 can induce an inflammatory condition in kidney by stimulating IL-6, CXCL8, and CCL2 [17]. IL-21 is another cytokine that is expressed at high levels by Th17 cells [18]. IL-6 was reported to be necessary for the differentiation of Th17 cells [19].

Paust *et al.* found that in comparison to nephritic wild-type mice, IL-17^−/−^mice developed less severe nephritis [20]. Liu *et al.* found that the Th17-Treg ratios increased along with elevated proteinuria and decreased albumin levels in patients with minimal change nephrotic syndrome. Huang *et al.* found that the number of CD4^+^CD25^+^ cells were significantly lower in IgAN cases than in the control group [11]. Wang *et al.* investigated mice infected by group A Streptococcus (GAS) intranasal (i.n.) and demonstrated that Th17 cells are the dominant T cells induced by i.n. infection. The association between Th17 response and GAS infection reveals a potential mechanism for detrimental autoimmune responses in humans [9]. The balance between Tregs and Th17 may influence pathology or disease outcome in autoimmune diseases [21]. In agreement with the study mentioned above, our results demonstrated that in comparison to controls, IgAN mice and HS-IgAN mice exhibited significant increase in renal Th17 cell numbers, Th17-related cytokines (IL-17, IL-21 and IL-6), as well as an obvious decrease in Treg numbers. Furhermore, the ratios of Th17 to Treg cell frequencies increased markedly in IgAN mice and reached the highest levels in HS-IgAN mice. These results suggested an imbalance in Th17/Treg function in IgAN mice, and that HS-infection can further break down the immunologic condition, consisting with what reported by Lin *et al* [22].

CCL20 is the founding member of the CC chemokine family [23] and the only chemokine known to interact with CCR6. The ligand-receptor CCL20-CCR6 plays a role in skin and mucosal surface under homeostatic and pathological conditions [24]. Both human and mouse Th17 cells can produce CCL20 [25], [26]. CCL20 promote migration of Th17 cells and Tregs in a CCR6-dependent manner. A lack of CCR6 in Th17 cells inhibits the recruitment of both Th17 and Tregs into inflammatory tissues [26], [27]. Consistently, Turner, *et al.* found that CCR6 deficiency reduced infiltration of Th17 cells in the setting of glomerulonephritis, which suggests that CCR6 mediates renal recruitment of Th17 cells [28].

Our results showed that the pathological features of CCL20-treated mice were greatly diminished, and the numbers of renal Th17 cells, as well as Th17-related cytokines (IL-17, IL-21, IL-6 and CCL20) were decreased compared with IgAN mice and HS-IgAN mice. The ratios of Th17 to Treg cell frequencies were also decreased. The decreased concentration of CCL20 indicated that CCL20-directed agents can partially reverse the broken immunologic status of Th17/Treg. Taken together, our data suggest that HS may induce the exacerbation of kidney damages through the CCL20/CCR6 response to the effect of Th17 cells in the pathogenesis of IgAN.

In summary, our study showed that a Th17/Treg functional imbalance in IgAN, and α-HS may worsen kidney damage in IgAN through CCL20 response to the effect of Th17 cells. However, in this process, whether other factors regulate Th17 cells remains to be clarified.

## Materials and Methods

### Ethics Statement

This study was carried out in accordance with the recommendations from the Guide for the Care and Use of Laboratory Animals published by the National Institutes of Health. The protocol was approved by the animal experimental ethics committee, Hunan Province (Permit Number: 20110003). All efforts were made to minimize animal suffering.

### Experimental Animals and Treatment

Six-week-old female BALB/c mice (20±2 g) were provided by Experimental Animal Center of Central South University (Changsha, Hunan, China). All animals were raised under the ideal temperature and humidity and specific pathogen-free conditions (six mice per cage). Thirty mice were randomly divided into five groups (n = 6 per group): control mice (control), IgAN mice (IgAN), HS-infected IgAN mice (HS-IgAN), CCL20-treated IgAN mice (CCL20-IgAN), and CCL20-treated HS infected IgAN mice (CCL20-HS-IgAN). IgAN model was induced by intragastric gavage BSA (Roche USA) acidified water (800 mg/kg body weight) every other day, subcutaneous injection of CCl_4_ and castor oil (mixed at the ratio of 1 to 5) (0.1 ml) once a week, combined with intraperitoneal injection (0.08 ml) biweekly, intravenous injection of LPS (Sigma, USA) (50 ug) twice at week six and week eight. For IgAN group, IgAN mice were received no treatment. For CCL20-IgAN group, IgAN mice were sensitized by intraperitoneal injection of anti-CCL20 (abcam, USA) (100 ug per mouse). For HS-IgAN group, IgAN mice were subject to the intranasal infection of live alpha-hemolytic streptococcus (α-HS) at the 10^th^ week. For CCL20-HS-IgAN group, IgAN mice were first treated with CCL20 antibody, followed by α-HS infection 24 h later, using the same regimen as described above. Controls received an equal amount of distilled water. α-HS was isolated from human tonsils and inoculated intranasal with a dose of 2×10^8^ CFUs in 10 µL PBS per mouse (5 µL per nostril) [9]. All mice were terminated at 11^th^ week after administration of HS and/or CCL20 antibody for kidney and lung harvest.

### Functional Studies

All the mice were housed in metabolic cages individually for 24 hours to collect urine samples. ACR was detected by standard laboratory methods.

### Morphological Changes Examination

The renal tissues were fixed in 4% paraformaldehyde and serially cut after embedded in paraffin. Tissue sections (4 µm in thickness) were stained with hematoxylin and eosin (HE) and periodic acid-Sachiff reagents. The lungs were processed similarly and stained with HE. The stained kidney and lung sections were examined and analyzed by a renal pathologist (Q. Z.) under a light microscope.

The percentage of abnormal glomeruli was evaluated by examining abnormalities at least in 50 glomeruli per mouse [29]. The abnormal glomeruli included segmental proliferation, mesangial matrix expansion, capillary wall thickening, glomerular hypercellularity, hyalinosis, crescent formation and fibrinoid necrosis.

### Immunofluorescence Analyses

For immunofluorescence analyses, renal tissues of mice were cut into frozen slices and fixed in acetone for 1 minute. After fixation, 5% normal goat serum in PBS (pH 7.4) was used to block nonspecific protein binding sites. IgA in renal tissues were detected with Fluorescein-labeled goat anti-mouse IgA (Santa Cruz: sc-3692).

### Transmission Electron Microscopy

The renal tissues of the mice were fixed with 2.5% glutaraldehyde in 0.1 M cacodylate buffer (pH 7.2). Three hours later, specimens were placed in 2% OsO_4_ for 2 h. Next they were hydrated in a decreasing series of ethanol solutions and embedded in Epon-Araldite. The specimens were cut into ultrathin sections (70 nm) and stained with uranylacetate and lead citrate. The samples were examined by an H-7700 transmission electron microscope (Hitachi, Japan).

### Leukocyte Isolation of Renal Tissue

For leukocyte isolation, unilateral kidneys of the mice were minced completely and processed with 0.4 mg/ml collagenase D (Roche) and 0.01 mg/ml DNase I in Dulbecco modified Eagle medium (DMEM; Hyclone) supplemented with 10% heat-inactivated fetal calf serum (FCS, Gibco) at 37°C for 45 minutes [28]. Cell suspensions were filtered through a series of nylon meshes at 70- and 40-µm and washed with PBS (Solarbio). Leukocyte-enriched cell suspension was obtained by Percoll density gradient (70% and 40%) centrifugation. Before flow cytometry, the viability of the cells was assessed by trypan blue staining.

### Flow Cytometry

Isolated leukocytes of mice were equally distributed into tubes and stained with fluorochrome-labeled antibodies specific for CD3 (PE-Cy5; eBioscience; USA) and CD4 (FITC; eBioscience; USA) for 30 minutes at 4°C. Normal mouse serum (Sigma) was used to block unspecific staining before antibody incubation. Staining of intracellular IL-17 was performed as follows [20]. Isolated renal leukocytes were suspended in RPMI 1640 (Gibco) with 10% FCS and activated by phorbol 12-myristate 13-acetate (PMA, 50 ng/ml; Sigma) and ionomycin (1 µg/ml; Sigma) in an incubator (37°C, 5% CO_2_) for 5 hours. After 30 minutes of incubation, Brefeldin A (3 µg/ml; eBioscience) was added. The cells were washed and stained by cell surface markers several steps, and then incubated in Cytofix/Cytoperm (eBioscience; USA) at 4°C for 30 minutes to permeabilize cell membranes. Then intracellular IL-17 was stained by rat anti-mouse IL-17 antibody (PE; eBioscience). Staining of Foxp3 was performed according to the manufacturer's instructions. Isolated renal leukocytes were incubated with CD4 (FITC; eBioscience; USA) and CD25 (APC; eBioscience; USA) in the dark at 4°C for 30 minutes and then dealt with Fix/Perm buffer (eBioscience; USA) for fixation and permeabilization, followed by staining of anti-mouse Foxp3 antibody (PE; eBioscience; USA) for 30 minutes. Then the cells were analyzed with a Becton Dickinson FACS calibur system using the Cell Quest software.

### Enzyme-Linked Immunosorbent Assay (ELISA)

The levels of IL-17A, IL-21, IL-6, and CCL20 in kidneys of mice were quantified by ELISA kits and performed according to the manufacturer's instructions (USCN, China). Immunoreactivity was determined by an ELISA reader at 450 nm.

### Statistical Analysis

All data are expressed as mean±standard deviation (x ± SD), and analyzed by multiple comparison tests and one-way analysis of variance (ANOVA). In case of multiple comparisons, one-way ANOVA and LSD-t test were used. Statistical significance was set at *P*<0.05. Statistical analyses were performed using SPSS 17.0 software.
